# Novel candidate taxa contribute to key metabolic processes in Fennoscandian Shield deep groundwaters

**DOI:** 10.1093/ismeco/ycae113

**Published:** 2024-09-23

**Authors:** Mark Dopson, Maryam Rezaei Somee, Carolina González-Rosales, Lauren M Lui, Stephanie Turner, Moritz Buck, Emelie Nilsson, George Westmeijer, Kamal Ashoor, Torben N Nielsen, Maliheh Mehrshad, Stefan Bertilsson

**Affiliations:** Centre for Ecology and Evolution in Microbial Model Systems (EEMiS), Linnaeus University, 39231 Kalmar, Sweden; Centre for Ecology and Evolution in Microbial Model Systems (EEMiS), Linnaeus University, 39231 Kalmar, Sweden; Centre for Ecology and Evolution in Microbial Model Systems (EEMiS), Linnaeus University, 39231 Kalmar, Sweden; Molecular Ecosystems Biology Department, Environmental Genomics and Systems Biology Division, Lawrence Berkeley National Laboratory, Berkeley, CA 94720, United States; Centre for Ecology and Evolution in Microbial Model Systems (EEMiS), Linnaeus University, 39231 Kalmar, Sweden; Department of Forest Mycology and Plant Pathology, Swedish University of Agricultural Sciences, P.O. Box 7050, 75005 Uppsala, Sweden; Department of Aquatic Sciences and Assessment, Swedish University of Agricultural Sciences, P.O. Box 7050, 75005 Uppsala, Sweden; Centre for Ecology and Evolution in Microbial Model Systems (EEMiS), Linnaeus University, 39231 Kalmar, Sweden; Centre for Ecology and Evolution in Microbial Model Systems (EEMiS), Linnaeus University, 39231 Kalmar, Sweden; Centre for Ecology and Evolution in Microbial Model Systems (EEMiS), Linnaeus University, 39231 Kalmar, Sweden; Molecular Ecosystems Biology Department, Environmental Genomics and Systems Biology Division, Lawrence Berkeley National Laboratory, Berkeley, CA 94720, United States; Department of Aquatic Sciences and Assessment, Swedish University of Agricultural Sciences, P.O. Box 7050, 75005 Uppsala, Sweden; Department of Aquatic Sciences and Assessment, Swedish University of Agricultural Sciences, P.O. Box 7050, 75005 Uppsala, Sweden

**Keywords:** deep biosphere, metagenomics, metatranscriptomics, Candidatus

## Abstract

The continental deep biosphere contains a vast reservoir of microorganisms, although a large proportion of its diversity remains both uncultured and undescribed. In this study, the metabolic potential (metagenomes) and activity (metatranscriptomes) of the microbial communities in Fennoscandian Shield deep subsurface groundwaters were characterized with a focus on novel taxa. DNA sequencing generated 1270 de-replicated metagenome-assembled genomes and single-amplified genomes, containing 7 novel classes, 34 orders, and 72 families. The majority of novel taxa were affiliated with *Patescibacteria*, whereas among novel archaea taxa, *Thermoproteota* and *Nanoarchaeota* representatives dominated. Metatranscriptomes revealed that 30 of the 112 novel taxa at the class, order, and family levels were active in at least one investigated groundwater sample, implying that novel taxa represent a partially active but hitherto uncharacterized deep biosphere component. The novel taxa genomes coded for carbon fixation predominantly via the Wood–Ljungdahl pathway, nitrogen fixation, sulfur plus hydrogen oxidation, and fermentative pathways, including acetogenesis. These metabolic processes contributed significantly to the total community’s capacity, with up to 9.9% of fermentation, 6.4% of the Wood–Ljungdahl pathway, 6.8% of sulfur plus 8.6% of hydrogen oxidation, and energy conservation via nitrate (4.4%) and sulfate (6.0%) reduction. Key novel taxa included the UBA9089 phylum, with representatives having a prominent role in carbon fixation, nitrate and sulfate reduction, and organic and inorganic electron donor oxidation. These data provided insights into deep biosphere microbial diversity and their contribution to nutrient and energy cycling in this ecosystem.

## Introduction

The deep biosphere is defined as the life below the Earth’s surface and is estimated to host 12%–20% of the total biomass on Earth [1, 2]. These microorganisms (along with viruses) are active and viable in groundwaters down to several kilometers (e.g. 2.8 km in the Mponeng mine, South Africa) below the Earth’s surface [1, 3–9]. Due to the prevailing low nutrient and energy availability, water-bearing bedrock fractures in the terrestrial deep biosphere typically feature low cell densities [10]. The understanding of diversity and metabolic capabilities of the vast deep biosphere microbiome remains constrained by barriers to study them. Access points to observe and sample these microbiomes are limited to boreholes, mines, or subsurface laboratories, making the deep biosphere one of the least understood biomes on Earth.

The “great plate count anomaly” revealed that the majority of the Earth’s microbial diversity remains uncultured [11]. The use of nucleic acid sequencing has aided investigation of this uncultured majority [12], e.g. community 16S ribosomal RNA gene sequences revealing that all major ecosystems except the human body are dominated by uncultured species [13]. In addition, assembled genomes from community DNA [14–16] and single-cell amplified genomes [12] provide an updated view of the tree of life. Omics studies have been applied to shallow groundwaters [17], geysers [18], mines [19], and deep groundwaters of the Fennoscandian Shield [10, 20, 21], revealing an impressive diversity of candidate and unknown taxa at different ranks. As many microbial species are not yet axenically cultured under laboratory conditions, efforts have been made to standardize microbial classification using genomic data rather than an axenic culture as type [22, 23]. Few validly described species have been cultured from Fennoscandian Shield deep groundwaters [24–26], whereas large-scale omics analysis has recovered more than 568 genome species (operational definition of species with ≥95% average genome nucleotide identity [20]). Additionally, widespread genome features of microbes inhabiting this ecosystem highlight the importance of biological interactions in sustaining many taxa [20], a factor that further decreases the possibility to obtain axenic cultures for taxa dependent on obligate interactions and metabolic dependencies. This is especially relevant for representatives of DPANN and *Patescibacteria* (previously defined as candidate phyla radiation bacteria) that encompass 30% of the recovered genome species in deep Fennoscandian Shield groundwaters [20].

The typically extreme conditions in the terrestrial deep biosphere, such as low energy and nutrient availability, select for a mixture of long-term indigenous taxa. In parallel, there are infiltrating microbes from the surface, including those that are able to adapt to this environment, such as *Patescibacteria*, *Pseudomonadota* (formerly *Proteobacteria*), and *Epsilonbacteraeota* [27], while non-viable cells appear to be rapidly degraded [28]. Compared to surface biomes that have rapid fluxes in typically light, temperature, and nutrient availability, geochemical conditions in the deep biosphere are recorded to be relatively stable for at least 15 years [21], and the energy available is generally low compared to principally photosynthetically-driven surface biomes [29]. Despite the isolated and stable conditions, dynamics in the microbial community composition can occur over multiple years with the most dynamic changes evidenced in shallow compared to deeper groundwaters [30]. Carbon and energy sources in Fennoscandian Shield oligotrophic deep groundwaters are suggested to be via infiltration of autochthonous surface species that subsequently die (termed necromass [28]), geogenic hydrogen and carbon dioxide [31], and surface-derived recalcitrant dissolved organic matter [32]. Analyses of deep subsurface groundwaters in the Fennoscandian Shield have revealed a shared “core” microbial community suggested to have adaptations to the low carbon and energy conditions of this ecosystem [20]. These adaptations (reviewed in [33]) include a small cell size with the potential to pass through a 0.2 μm pore size membrane typically used for cell capture [34], streamlined genomes calling for metabolite exchange and symbiotic interactions [35], and an “episodic lifestyle” in response to intermittent availability of nutrients and energy sources [20]. Despite these previous studies, further inquiries into the diversity of subsurface groundwaters in the Fennoscandian Shield continue to reveal many novel taxa for which the biological roles remain to be elucidated.

While the deep biosphere is the largest biome on Earth, the vast majority of its microbial diversity remains undescribed, and consequently, their contribution to ecosystem function remains overlooked. To address this knowledge gap, this study investigated novel microbial candidate taxa from deep subsurface groundwaters in the Äspö Hard Rock Laboratory (Äspö HRL; Sweden) and Olkiluoto Island (Finland) using a combined long- and short-read metagenomic and short-read metatranscriptomic approach. The questions addressed include elucidating the metabolic potential of these novel deep subsurface populations and comprehensive mapping of their metabolic role in the community.

## Materials and methods

### Sampling sites

**Figure 1:**
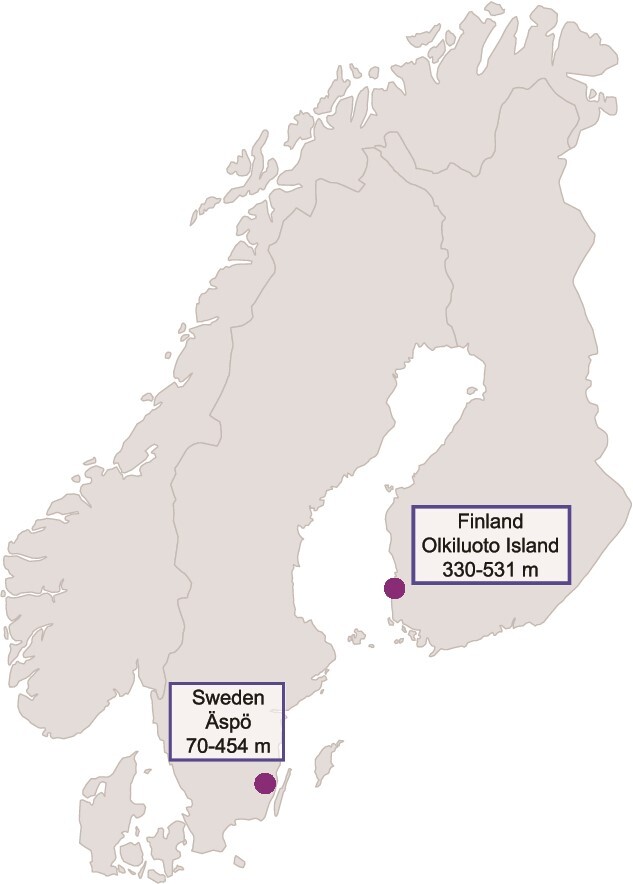
Map of the sampling sites at the Äspö HRL and Olkiluoto Island. Figure generated in the ggplot2, maps, and sf packages in R.

Metagenomic and metatranscriptomic datasets (Supplemental Table S1 and Fig. 1) were generated from the microbial community sampled from the Äspö HRL underground facility managed by the Swedish Nuclear Fuel and Waste Management Company (SKB). Äspö HRL consists of a 3.6 km tunnel extending to ~460 m below sea level (mbsl; Lat N 57° 26′ 4″, Lon E 16° 39′ 36″). Further metagenomic datasets were generated from Olkiluoto Island on the Finnish Baltic Sea coast (Lat N 61° 14′ 31″, Lon E 21° 29′ 23″) with boreholes to depths greater than 500 mbsl operated by Posiva Oy. The Äspö HRL tunnel provides access to investigate deep microbial life in 1.8 Ga old Proterozoic crystalline bedrock of the Fennoscandian Shield (also termed the Baltic Shield; area of the Earth’s crust encompassing large parts of Norway, Sweden, Finland, and Russia [31]), with the Äspö HRL geology, hydrology, and chemistry having been previously reported [36–38]. Olkiluoto Island drillholes and tunnels penetrate Precambrian metamorphic rocks with igneous rocks as previously described [39], where the drawdown of waters can result in some mixing, but there was no evidence of oxygen infiltration.

### Cell capture from groundwaters

A portion of the community nucleic acids from planktonic and biofilm cells captured from the various groundwaters included in this investigation were previously published [20, 21, 40–42]. Further metagenomes and metatranscriptomes were generated for this study according to the previously described methods (Table 1) plus for nanopore sequencing in Supplementary File S1. Briefly, planktonic microbial cell fractions for metagenomes were captured by flushing stagnant water from the borehole to ensure a representative sample was obtained, and then connecting a high-pressure holder containing a 0.1 μm filter and allowing water to pass through the filter under *in situ* temperature and pressure. The filters were then aseptically collected before flash freezing in liquid nitrogen and stored at −80°C until processing. In addition, biofilm cells were collected in flow cells containing solid support directly attached to the boreholes, where the groundwater was allowed to flow under *in situ* temperature and pressure before opening the vessels for cell recovery [40]. Finally, cells for community RNA transcripts were captured on 0.1 μm filters under *in situ* temperature and pressure, followed by directly flash freezing in liquid nitrogen and storage at −80°C [41]. The DNA/RNA extraction method for each sample and sequencing platform is provided in Supplementary Table S1.

**Table 1:** Details of the nucleic acid sequencing used in this study and not published in the previous iteration of the FSG [20].

**Designation**	**Borehole**	**Nucleic acid**	**Platform**	**Depth (mbsl)**	**Metagenome/−transcriptome accession numbers (NCBI)**
AHRL-69.4	KR0015B	DNA	Illumina NovaSeq	69.4	SRR26275512
			Oxford Nanopore		SRR27925880
		RNA	Illumina NextSeq		SRR26275508
AHRL-200.6	SA1420A	DNA	Illumina NovaSeq	200.6	SRR26275511
		RNA	Illumina NextSeq		SRR26275507
AHRL-281.6	SA2074A	DNA	Illumina NovaSeq	2817	SRR26275510
AHRL-345.0	SA2600A	DNA	Illumina NovaSeq	345.0	SRR26275509
		RNA	Illumina NextSeq		SRR26275506

### Bioinformatic analyses

Deep groundwater metagenomes (57 samples) were captured from the Äspö HRL (KR0015B, KF0069A01, SA1229A, SA1420A, SA2074A, KA2198, SA2600A, KA3105-4, and KA3385A) and Olkiluoto Island (KR_11, KR_13, and KR_46) boreholes (metadata and a map of the Äspö HRL boreholes are previously published [10, 20, 21]) with details given in Table 1, Supplementary File S1, and Supplementary Table S1. Detailed bioinformatic methods are provided in Supplementary File S1. Briefly, Fennoscandian Shield genome (FSG) metagenomic datasets were quality-checked and trimmed by Trimmomatic [43] and then assembled using MEGAHIT [44]. Contigs ≥2 kb were binned using MetaBAT2 [45], genome quality assessed via CheckM [46], and taxonomy assigned using GTDB-tk [47]. Single-cell amplified genomes were assembled and quality checked as previously explained [20]. MAGs/SAGs were de-replicated based on similarity thresholds using fastANI by mOTUlizer [48]. This resulted in 112 representative MAGs/SAGs that were denoted as unclassified by GTDB-tk at the family level and above that were selected for further analysis and were assigned unique codes based on their origin with corresponding accession numbers given in Supplementary Table S2. Recovered MAGs/SAGs were metabolically annotated via the METABOLIC tool in community and genome-level modes [49]. The abundance of 112 candidate MAGs/SAGs in each trimmed metagenome and metatranscriptome samples was calculated by CoverM (v.0.6.1) using TPM as the normalization method, with further details available in Supplementary File S1.

## Results and discussion

The present study contributed new long- and short-read metagenome (13 samples from 4 boreholes) and short-read metatranscriptome (9 samples from 3 boreholes) sequence data from planktonic cells in Äspö HRL deep subsurface groundwaters (Table 1). This resulted in 1.15 TB of metagenomic data that were combined with previously published sequencing results [20] to enable reconstruction of 2295 MAGs and SAGs with ≥50% completeness and ≤5% contamination. These MAGs/SAGs were de-replicated into 1270 representative assembled genomes, from which novel taxa identified according to taxonomic assignment of the genome taxonomy database (GTDB) were selected for further analyses.

### Number and abundance of novel FSG taxa

**Figure 2:**
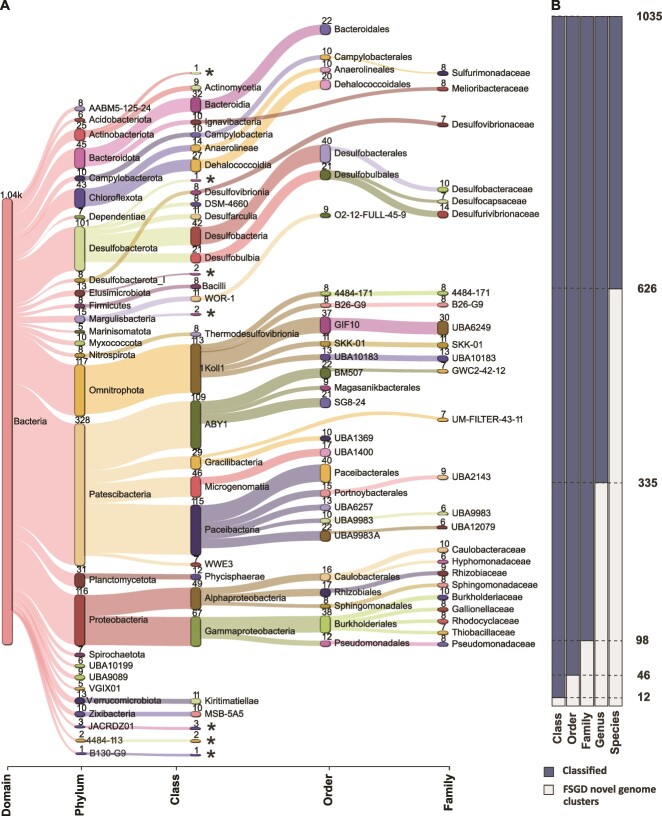
Taxonomic profile information of FSG bacterial metagenome assembled genomes (MAGs) and single amplified genomes (SAGs). (A) Distribution of MAGs/SAGs in different phylogenetic levels, with the top 25 taxa at each level. Each number indicates the number of cluster MAGs/SAGs in that taxonomic level. Novel candidate classes marked with an asterisk. (B) Classified/unclassified number of MAGs/SAGs by GTDB-tk in each taxonomic level.

**Figure 3:**
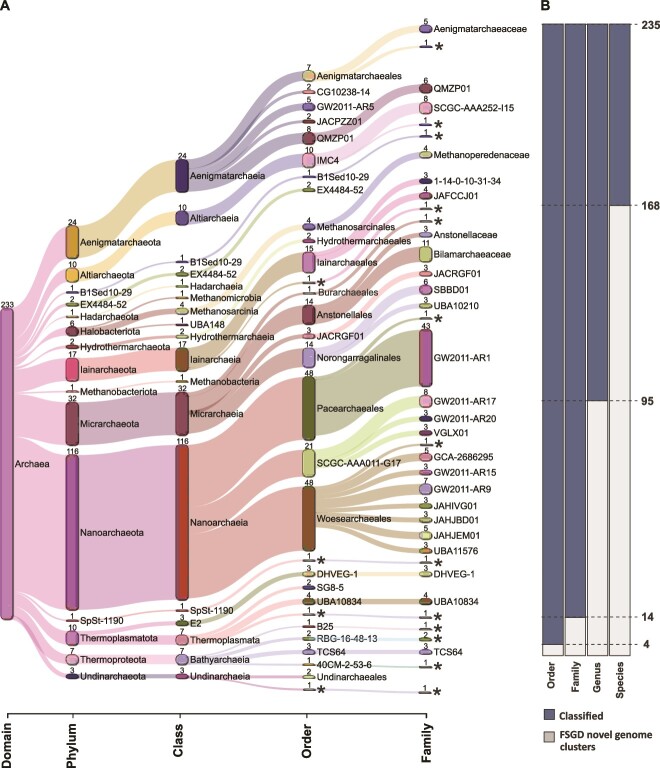
Taxonomic profile information of FSG archaeal metagenome assembled genomes (MAGs) and single amplified genomes (SAGs). (A) Distribution of MAGs/SAGs in different phylogenetic levels, with the top 25 taxa at each level. Each number indicates the number of cluster MAGs/SAGs in that taxonomic level. Novel candidate classes marked with an asterisk. (B) Classified/unclassified number of MAGs/SAGs by GTDB-tk in each taxonomic level.

While the updated FSG did not contain any novel phyla, there were 12 de-replicated representative bacterial MAGs/SAGs assigned to seven novel candidate classes, 46 representative MAGs/SAGs in 34 novel candidate orders, 98 MAGs/SAGs in 72 novel candidate families, 335 MAGs/SAGs in 230 novel candidate genera, and 626 MAGs/SAGs in 463 novel candidate species (Fig. 2 and Supplementary Table S2). Analogously, the genomes contained novel archaea, with four de-replicated representative archaeal MAGs assigned to separate candidate orders, 14 MAGs in 13 candidate families, 95 MAGs in 65 candidate genera, and 168 MAGs in 115 candidate species (Fig. 3 and Supplementary Table S2). The high number of previously undescribed taxa in the FSG were in line with the existing view of the deep biosphere as an untapped reservoir of biological novelty [17]. The FSG encompasses groundwaters of differing depths, ages, and sources from two sites on either side of the Baltic Sea, which exhibit a greater microbial diversity as compared to other Fennoscandian Shield [50], North American [51], and South African [52] groundwaters greatly dominated by a single taxa. However, the factors controlling the large differences in worldwide deep groundwater diversities have not been elucidated.

**Figure 4:**
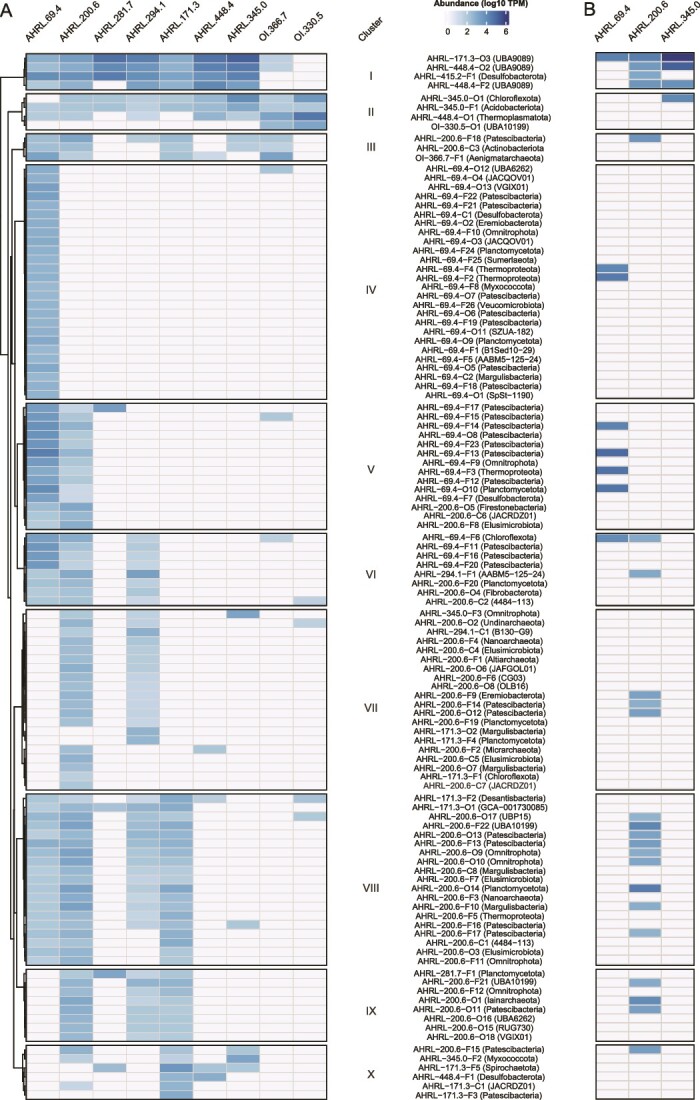
Mapping metagenomic (A) and metatranscriptomic (B) reads against novel FSG taxa at the levels of class, order, and family. (A) Average of the abundance (log_10_ of TPM) of 112 novel taxa in groundwater samples from Äspö HRL (AHRL) and Olkiluoto Island (OI). OI-528.7 (borehole OL-KR46) was not included as no novel taxa at family or above was detected in this groundwater. (B) Average of the RNA transcript abundance (log_10_ of TPM) of 112 novel taxa in selected Äspö HRL groundwaters (no data for OI boreholes were available).

The bacterial phyla with the highest number of novel MAGs/SAGs at the levels of class, order, and family was *Patescibacteria* with 27 representatives, followed by *Planctomycetota* (8 MAGs/SAGs), *Omnitrophota* (7 MAGs/SAGs), Margulisbacteria plus *Elusimicrobiota* (both 5 MAGs/SAGs), and *Desulfobacterota* (4 MAGs/SAGs). *Patescibacteria* comprise a large percentage of the bacterial phylogenetic diversity [14, 53] and have been extensively identified in near-surface and deep groundwaters [14, 17]. The *Patescibacteria* are suggested to infiltrate groundwaters due to their high tendency of being exported from deep soil horizons along with streamlined genome features that support a simple fermentative lifestyle that enable them to survive in low carbon and energy conditions [54]. The *Omnitrophota* have been identified in the deep terrestrial biosphere at the DeMMO site, South Dakota [55]. This taxa is also adapted to the deep biosphere by having a small cell size and reduced genomes, even if various lineages code for assorted respiration processes for energy conservation (e.g. dissimilatory nitrite reduction plus sulfur and iron oxidation [55]) and gene clusters indicative of symbiotic lifestyles [56]. The *Margulisbacteria* were also identified from shallow suboxic/anoxic groundwater, where the majority of the community was suggested to lack genes coding for complete metabolic pathways [17]. The *Elusimicrobiota* were originally defined as endosymbionts [57], while subsequently described free-living groundwater populations are metabolically diverse [58]. Finally, the *Desulfobacterota* are known to populate anaerobic groundwaters [59]. The novel archaeal taxa were affiliated to *Thermoproteota* (5 MAGs/SAGs) and *Nanoarchaeota* (2 MAGs/SAGs). The recently described *Candidatus Methanodesulfokores washburnensis* from the *Thermoproteota* has been identified in the Pennsylvania, USA, deep subsurface [60] and is capable of both methanogenesis and sulfate reduction that makes it potentially possible to perform anaerobic oxidation of methane within cellular boundaries [61]. Based upon a single transcript being represented for the ORFs in each of the MAGs/SAGs with a TPM value >100, 89 out of the 112 novel taxa were considered to be active in at least one groundwater. Furthermore, when plotting the same metatranscriptomes as transcripts per million (TPMs), the low transcript numbers were rounded to zero, such that 30 out of the 112 novel taxa were also considered as active (Fig. 4 and Supplementary Table S3). This supports previous studies in the Fennoscandian Shield showing a broad active community in Äspö HRL [41, 42, 62] and South African [5] groundwaters.

Clustering of the novel classes, orders, and families based on relative abundances and incidence (rather than phylogeny) in selected Äspö HRL and Olkiluoto Island sites revealed four representative MAGs/SAGs (cluster I) almost ubiquitously present across the investigated Fennoscandian Shield groundwaters (Fig. 4 and Supplementary Table S3). This cluster included two novel orders (AHRL-171.3-O3 and AHRL-448.4-O2) and a novel family (AHRL-448.4-F2) from the UBA9089 phylum that have solely been identified from groundwaters [20, 63] plus a novel *Desulfobacterales* family (AHRL-415.2-F1) [64]. The three UBA9089 phylum MAGs/SAGs were also highly represented in the Äspö HRL metatranscriptomes, particularly in the AHRL_345.0 groundwater. In contrast, AHRL-415.2-F1 RNA transcripts were only present in AHRL_200.6.

Clusters II and III (four and three MAGs/SAGs, respectively) were also mostly distributed across the investigated groundwaters with particularly high representation in the shallowest Olkiluoto Island groundwater for cluster II. Cluster II MAGs/SAGs included the novel order OI-330.5-O1 of the UBA10199 phylum suggested to have streamlined genomes [65], the AHRL-345.0-F1 family from the RPQK01 order in the *Acidobacteriota*, and AHRL-345.0-O1 attributed to the heterotrophic and obligatory anaerobic *Anaerolineae* (*Chloroflexota* phylum) [66]. Cluster III MAGs/SAGs included AHRL-200.6-F18 from the *Portnoybacterales* (*Patescibacteria*) that was also active in the AHRL_200.6 groundwater.

All novel FSG MAGs/SAGs from cluster IV were solely identified in the AHRL.69.4 groundwater except for AHRL-69.4-O12 within the UBA6262 phylum that was also identified in the OI.366.7 groundwater. These included AHRL-69.4-O5 from the ABY1 class (*Patescibacteria*) reported to contain ultra-small representatives that actively replicate in groundwaters [67]. Members of the *Patescibacteria* are enriched in aquifer sediments, where they largely exhibit a surface-attached lifestyle. This preference for surface attachment is attributed to higher environmental stability, greater access to nutrients, and proximity of putative symbiotic partners as compared to being free-living and planktonic [67]. Nevertheless, previous work has demonstrated that the Fennoscandian Shield early-stage biofilm formation can be attributed to autotrophic and diazotrophic populations, including the *Thiobacillus* genus, while *Patescibacteria* are suggested to attach only after the biofilm has formed [40, 68]. Further FSG MAGs/SAGs in this cluster included a novel family (AHRL-69.4-F21) in *Veblenbacterales* (*Patescibacteria*), an order containing other representatives identified in a shallow aquifer [17]; a novel family (AHRL-69.4-F24) in the order H5-PLA8 from the *Planctomycetota*; a *Desulfobacterota* class (AHRL-69.4-C1); a novel family (AHRL-69.4-F26) within the class *Methylacidiphilales* suggested to feature aerobic and acidophilic representatives [69]; and a novel *Xenobia* order (AHRL-69.4-O2) from the *Eremiobacterota* of which some orders are facultative anaerobes [70]. Of the cluster IV MAGs/SAGs, AHRL-69.4-F2 and AHRL-69.4-F4 were identified as having significant numbers of RNA transcripts in the AHRL.69.4 groundwater. Both these novel archaeal families were from the *Bathyarchaeia* (*Thermoproteoata*) that encode various one-carbon utilization strategies, including methane oxidation [71].

MAG cluster V was ubiquitously represented in the Äspö HRL AHRL.69.4 and AHRL.200.6 boreholes but rare in the other groundwaters. The MAGs/SAGs in this cluster included seven aligning with the *Patescibacteria*, of which AHRL-69.4-F13 and AHRL-69.4-F14 (both order JAACEG01) were also active in the AHRL.69.4 borehole. In addition, AHRL-69.4-F3 (*Bathyarchaeia* order RBG-16-48) and *Planctomycetota* AHRL-69.4-O10 were active in this groundwater.

The remaining MAG/SAG clusters VI–X included novel taxa identified in several of the Äspö HRL groundwaters but again were rare in Olkiluoto. These included the *Omnitrophota* family AHRL-200.6-F12 suggested to feature traits, such as being ultra-small and host-dependent to survive low carbon and energy conditions [56] and a novel *Pacearchaeales* taxa at family level (AHRL-200.6-F3) belonging to *Nanoarchaeota* DPANN archaea [12]. Co-occurrence analysis for populations identified in groundwaters of varying depths suggested the order *Pacearchaeales* from the *Nanoarchaeota* were putative symbiotic partners of *Patescibacteria* [72]. The active novel taxa from these clusters were solely identified in groundwater AHRL.200.6 and included AHRL-200.6-O12 from the *Patescibacteria* class UBA1384 previously suggested to contribute to a New Zealand groundwater ultra-small prokaryote RNA transcript-based activity [67]; AHRL-200.6-F14 from the ABY1 class; the novel family AHRL-200.6-F10 from the *Margulisbacteria* [17]; and a *Patescibacteria* family AHRL-200.6-F17 previously suggested to lack genes for amino acid or nucleotide biosynthesis [53].

### Metabolic potential for carbon and nitrogen fixation

**Figure 5:**
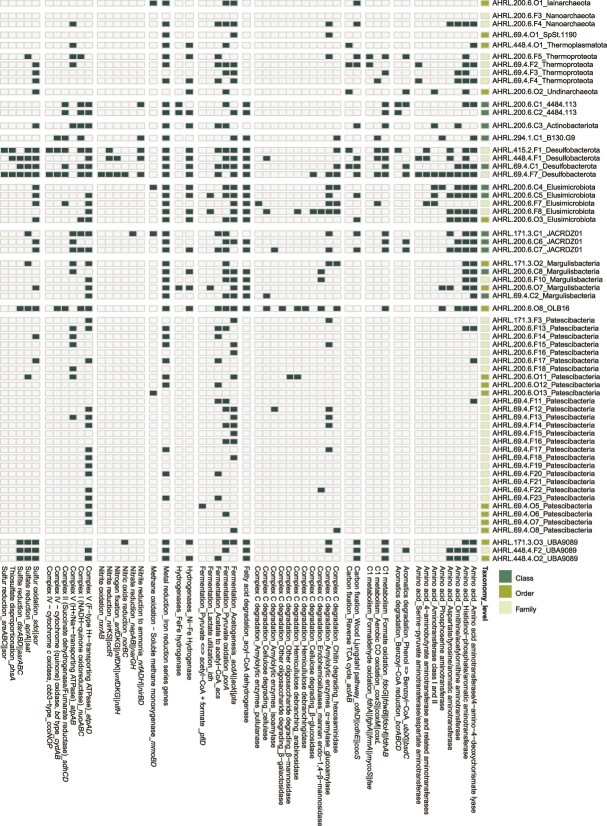
Presence of selected metabolic traits in a sub-set of the novel FSGs (full version of the figure is available in Supplementary Fig. S3). Certain categories, such as “Complex carbon degradation_Hemicellulose debranching,” that are made up of several individual genes (and consequently data lines) in Supplementary Fig. S3 have been amalgamated for space considerations. Presence is indicated by dark color and absence light (left). The rank of novel taxa is indicated on the right.

Autotrophic carbon fixation encoded in the novel classes, orders, and families of the FSG included the Wood–Ljungdahl pathway (37 out of the 112 novel MAGs/SAGs affiliated to 21 phyla) along with ten MAGs/SAGs from seven phyla carrying genes coding for the reverse TCA cycle (Fig. 5 and Supplementary Fig. S1). Genes encoding the Wood–Ljungdahl pathway were present in 6 out of the 7 *Omnitrophota*, 3 out of the 4 *Desulfobacterota*, all three UBA9089, 3 of the 5 *Elusimicrobiota*, both *Myxococcota* and VGIX01 (formerly *Candidatus Eisenbacteria* [17]), and 1 *Altiarchaeota* MAGs/SAGs. The presence of the Wood–Ljungdahl pathway has previously been reported for the *Omnitrophota* [56], *Desulfobacterota* plus UBA9089 [20], *Elusimicrobiota* [58], *Myxococcota* [73], VGIX01 [17], and *Altiarchaeota* [74]. This pathway is often prevalent in the deep biosphere (e.g. South Africa and USA [19, 75]), as it is favored by anaerobic populations close to the thermodynamic limits of life and requires strict anoxia [76]. In addition, genes coding for the reverse TCA cycle were identified in all three UBA10199, 2 out of 4 of *Desulfobacterota*, and a single MAG from the *Undinarchaeota*, *Altiarchaeota*, and *Thermoplasmatota*. This confirmed a previous report that both *Desulfobacterota* and UBA9089 use the reverse TCA cycle and conserve energy from sulfate reduction [77].

Nitrogen fixation potential was identified in three of the novel MAGs/SAGs, namely two out of four *Desulfobacterota* and one of the two *Myxococcota*. However, the novel order AHRL-69.4-O12 assigned to the previously reported diazotrophic phylum UBA6262 [78] lacked apparent genes for nitrogen fixation, potentially narrowing the metabolic versatility of this deep biosphere candidate taxa or that the genes were not assembled in the incomplete MAG. Nitrogen fixation is common in some terrestrial deep biosphere environments and has been reported in the Horonobe Underground Research Laboratory in Japan [79].

### Metabolic potential for oxidation of inorganic electron donors

Genes encoding electron donors that were potentially capable of supporting a lithotrophic lifestyle included sulfide oxidation (as represented by *fccB* and *sqr*) and sulfur oxidation (*sdo* and *sor*) that were present in one and 50 of the 112 novel MAGs/SAGs, respectively. The novel taxa encoding genes for sulfur oxidation included all four *Desulfobacterota* MAGs/SAGs, all three UBA9089 MAGs/SAGs, and one *Margulisbacteria* MAG/SAG (AHRL-200.6-O7). Margulisbacteria were previously identified as mediating sulfate/sulfite reduction [20, 59, 63], and these data suggested that the novel FSG MAG/SAG was able to carry out both the oxidative and reductive steps in sulfur cycling [80]. While *Desulfobacterota* encompass sulfate-reducing populations, the presence of sulfur oxidation genes also expands the metabolic repertoire of this phylum. While *Patescibacteria* are often considered to have simple fermentative lifestyles encoded by a reduced genome (e.g. *Saccharimonadia* [81]), the *Portnoybacterales* have been implicated in sulfur cycling [17] even if the novel AHRL-200.6-F18 family (belonging to the *Portnoybacterales*) lacked genes for sulfur oxidation. In contrast, the novel families AHRL-200.6-F17 and AHRL-200.6-F14 MAGs/SAGs (*Paceibacterales* and SC72 orders, respectively) contained genes for sulfur oxidation, suggesting several *Patescibacteria* lineages have a broader metabolic repertoire than simple fermentative processes and highlights the importance of investigating their underexplored diversity and metabolic potential.

Another potential electron donor is hydrogen, which can be produced by, e.g. water radiolysis and serpentinization. Hydrogen is consumed by respiratory or bi-directional Ni/Fe hydrogenases [82], such as group 1 (10 MAGs/SAGs), group 3c (23 MAGs/SAGs), and group 3abd (5 MAGs/SAGs), which were identified in 26 phyla and thereby clearly demonstrating that capacity for hydrogen oxidation is a common trait across these novel taxa. Hydrogen oxidation is reported in the Fennoscandian Shield subsurface [83], and MAGs/SAGs with these genes included the previously reported hydrogen oxidizers *Margulisbacteria* [17], *Desulfobacterota* [84], and RUG730 that was formerly classified as Elusimicrobium [85]. Hydrogen-dependent terrestrial deep biosphere environments have been reported from around the globe (e.g. [75, 86]) with its importance suggested to increase with depth from the surface (reviewed in [2]).

### Metabolic potential for oxidation of organic electron donors

An additional source of nutrients and energy in deep groundwater is necromass [28], which here is broadly defined as polymers and dissolved organic constituents of biogenic origin either from dead cells or excreted compounds produced by, e.g. fermentation (Fig. 5 and Supplementary Fig. S1). Processes related to polymer hydrolysis, amino acid use, and fermentation were present among the novel taxa that lacked other apparent energy acquisition mechanisms (all but two of the novel *Patescibacteria* and all the 4484-113, *Aenigmatarchaeota*, GCA−001730085, and *Verrucomicrobiota* phyla). Nevertheless, some of these taxa are known to be sustained by symbiotic interactions for life in deep groundwaters, e.g. compensating for gaps in their metabolic capacity, while concomitantly providing excreted metabolites to their symbiotic partners [20, 87, 88]. Among the novel taxa, certain phyla, such as *Planctomycetota*, UBP15, Sumerlaeota, *Chloroflexota*, and OLB16, featured an abundance of biopolymer degrading pathways for hydrolysis of chitin and carbohydrates. The presence of these pathways in the novel taxa suggested capacity to use and assimilate necromass in this environment. However, it should be noted that many key genes involved in heterotrophic utilization of these compounds can have other roles in the cell, including recycling of cellular materials also in autotrophs. A further pathway potentially involved in necromass utilization is degradation of the aromatic amino acid phenylalanine incorporating anaerobic aromatic degradation mediated by *bcrABCD* that was limited to five of the novel taxa, such as 4484-113, one *Chloroflexota*, and one *Desulfobacterota* MAG/SAG.

### Metabolic potential for methane oxidation

Methane is an additional relevant electron donor in deep biosphere habitats, with methanotrophy enabled by the *mmoBD* genes. These genes were present in eight of the novel taxa of MAGs/SAGs, including two MAGs/SAGs from the *Planctomycetota*, one MAG from the *Patescibacteria*, SZUA−182, JACRDZ01, *Iainarchaeota*, *Elusimicrobiota*, and AABM5−125−24. The identification of methanotrophy in, e.g. the *Patescibacteria* widens the previously recognized genetic capability of affiliated populations. Active anaerobic oxidation of methane has previously been reported for the Fennoscandian Shield deep subsurface and is potentially coupled to sulfur cycling [89].

### Metabolic potential for energy conservation via electron transport

Energy conservation can be achieved via nitrate reduction to nitrite enabled by *napAB*/*narGH* that were present in five MAGs/SAGs, or via nitrite reduction to ammonia enabled by *nrfADH*/*nirBD* present in nine MAGs/SAGs, and finally via denitrification enabled by *nirKS*/*octR* found in three MAGs/SAGs. These nitrogen reduction processes were highly represented in the *Desulfobacterota* novel lineages as previously described [90]. A further energy conservation pathway is the reduction of sulfur-(*sreABC*/*sor*) present in 2 MAGs/SAGs, sulfite-(*dsrABD*/*asrABC*) present in 12 MAGs/SAGs, and sulfate (*aprA*/*sat*) present in 23 MAGs/SAGs. These MAGs/SAGs included sulfate/sulfite/sulfur reduction in the four, three, and two *Desulfobacterota* MAGs/SAGs, respectively. In addition, sulfate/sulfite reduction was present in all three UBA9089 MAGs/SAGs, the single UBP15 plus OLB16 MAGs/SAGs (formerly *Candidatus Omnitrophica* [12]), and both *Myxococcota* MAGs/SAGs. Sulfate reduction has previously been reported for the *Desulfobacterota* [59], UBA9089 [20, 63], and OLB16 (*Ca. Omnitrophica*) [56]. Sulfate/sulfite/sulfur reduction is commonly suggested to be an important energy conservation method in the terrestrial deep biosphere [20, 21, 80]. Energy conservation can also be mediated by ferric iron [91] and manganese [92] reduction that was present in 90 of the novel MAGs/SAGs, although it remains uncertain whether these genes mediated electron transfer. Finally, while one of the three novel *Thermoproteota* AHRL-200.6-F5 could reduce sulfate, it was not suggested to be capable of methanogenesis as described for *Ca*. *Methanodesulfokores washburnensis* [61].

Electron transport via complexes I, II, and IV was common among the novel taxa, with the exception of, e.g. *Patescibacteria*, which have been reported as having an energy conservation predominantly via fermentation [87]. In contrast, one or both of the two ATPase complexes (F- and V-types) were also broadly present in fermenters, such as *Patescibacteria* (19 out of 27 MAGs/SAGs). The ability to produce an F-type ATPase has previously been reported for the cultured *Patescibacteria* (*Saccharibacteria*) *Southlakia epibionticum* [93]. Such ATPases can operate in reverse by pumping out protons to sustain a proton motive force, but this was considered less likely in *S. epibionticum*, where instead it is suggested to use the ATPase in pH homeostasis. However, it was unclear which of these potential roles the ATPase plays in the deep groundwater populations. An alternative electron transport system (among other functions of this complex) is the RNF complex that was present in 41 of the 112 novel taxa (based on the presence of at least three out of the six *rnf* genes), including two each of the *Desulfobacterota* and *Elusimicrobiota*, one of the two *Nanoarchaeota*, all the JACRDZ01, JAFGOL01, *Margulisbacteria*, *Omnitrophota*, and *Ca. Eisenbacteria* VGIX01 (Supplementary Fig. S2). The RNF complex was widely prevalent among the novel taxa as well as the deep biosphere community, suggesting it may act as a basal electron transport system that requires less energy than complexes I–IV, which could potentially be an adaptation to the deep groundwater milieu. For instance, the RNF complex is utilized for autotrophic CO_2_ reduction [77] at the “thermodynamic limit of life” [94].

### Metabolic potential for energy conservation via fermentation

In the absence of thermodynamically favorable electron acceptors, fermentation emerges as a feasible and widespread metabolic strategy for organic carbon degradation (e.g. in South Dakota, USA, groundwaters [55]). In accordance with this, the capacity for several fermentative pathways (e.g. acetogenesis, lactic acid fermentation, and pyruvate oxidation based upon their potential to contribute to fermentation pathways while alternative pathways for the putative fermentation genes were incomplete in that MAG/SAG) was detected across many of the novel taxa with the potential for production of both acetate via the Wood–Ljungdahl pathway and hydrogen via Fe/Fe hydrogenases. The hydrogen would then likely act as an electron donor for pathways, such as sulfate reduction [63] and methanogenesis [95], since high concentrations of fermentation products are unlikely to accumulate in low carbon and energy conditions. Additionally, oxidation of one-carbon fermentation products, such as formate and formaldehyde, was prevalent among the novel taxa except for representatives of the *Patescibacteria*, *Margulisbacteria*, *Elusimicrobiota*, and *Nanoarchaeota*. These fermentation products could also play a role in reciprocal metabolic partnerships in deep groundwater biomes [20].

### Community-level contribution of novel taxa to metabolism

The community-scale metabolic capacity of each borehole (expressed as a percentage) for different metabolic functions was calculated by the METABOLIC tool [49], via a metric called metabolic weight score (MW-score; Supplementary Fig. S3). To calculate the MW-score for each function, the coverage values of genomes containing that function are summed and normalized by the overall coverage of the function [49]. A higher MW-score indicates more frequently shared functions and their increased abundance in the microbial community in each borehole. Furthermore, the contribution percentage of each MAG to each metabolic module function is determined, providing insights into the relative contributions of different microbial groups.

For the entire community in each groundwater type, these included fermentation (≤9.9%), amino acid utilization (≤9.5%), complex carbon (≤8.9%), aromatic degradation (≤5.6%), and fatty acid degradation (≤8.0%), pointing at one of the main nutrient sources in FSG groundwater (necromass). Of the carbon fixation processes, the Wood–Ljungdahl pathway was most prevalent (≤6.4%) except for borehole Ol-366.7, where the Calvin–Benson–Bassham cycle (7.5%) was the dominant autotrophic process. Furthermore, the novel taxa contributed to the oxidation of lithotrophic electron donors, including sulfur species (≤6.7%) and hydrogen (≤8.6%). Finally, the novel taxa also contributed significantly to energy conservation via electron transport that included nitrate (≤4.2% for *napAB*) and sulfate (≤6.0%) reduction.

**Figure 6:**
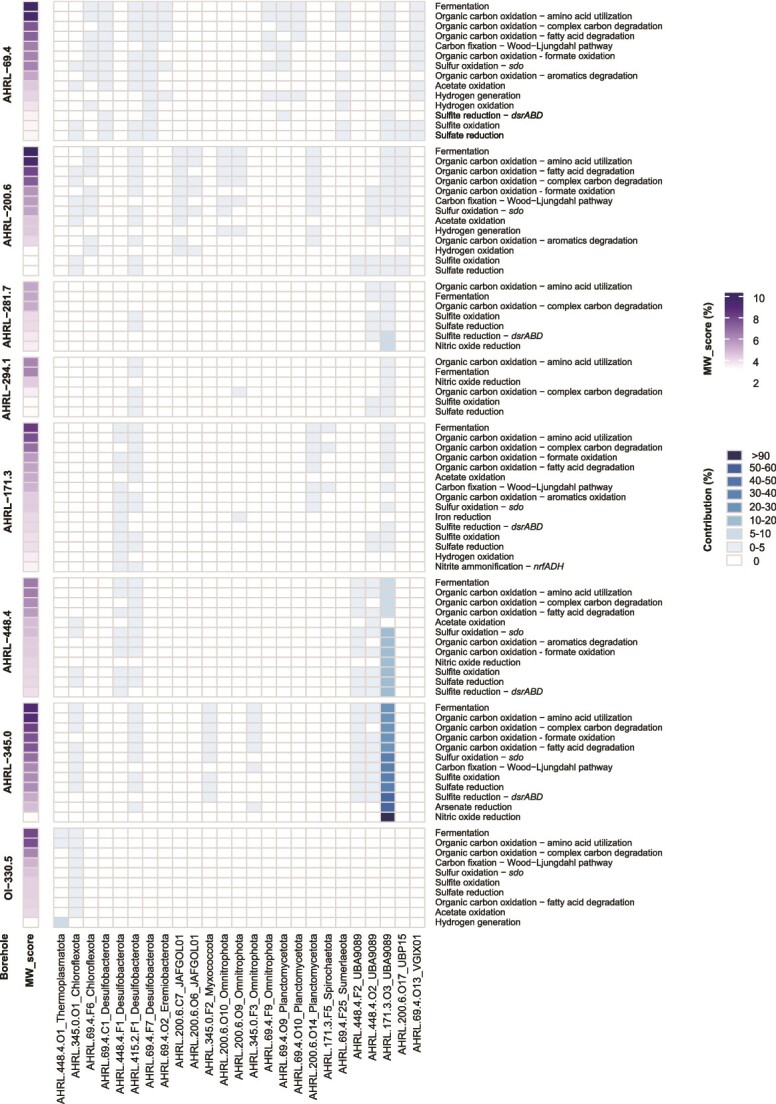
Contribution of selected novel taxa in functions with MW score ≥ 3 (MAGs with zero percent contribution are not shown) and the metabolic processes they contribute to. Borehole OI−366.7 that lacked any MAGs/SAGs contributing to the metabolisms was omitted. No contribution was observed between 60% and 89%.

Among the novel taxa, AHRL-171.3-O3, AHRL-448.4-O2, and AHRL-448.4-F2 MAGs/SAGs from the UBA9089 phylum strongly contributed to important metabolic functions in three Äspö HRL boreholes (Fig. 6 and Supplementary Table S4). For instance, the combined contribution of AHRL-171.3-O3 and AHRL-448.4-O2 in borehole AHRL−345.0 accounted for 91.9% of the total community’s nitric oxide reduction; 59.5% plus 51.4% of the total community’s sulfite and sulfate reduction; 50.1% of the total community’s carbon fixation by the Wood–Ljungdahl pathway; and 51.4% plus 45.5% of the total community’s sulfite and sulfur oxidation, respectively. In addition to the novel UBA9089, the AHRL-345.0-O1 MAG/SAG attributed to the *Anaerolineae* contributed e.g. an additional 3.2% each to the total AHRL−345.0 borehole community’s Wood–Ljungdahl pathway, sulfite oxidation, and sulfate reduction MW-scores, suggesting this groundwater was dominated by the two UBA9089 and one Chloroflexota novel taxa.

Other influential novel MAGs/SAGs contributing to the MW-scores included AHRL-448.4-O1 from the Thermoplasmatota contributing 6.1% of the hydrogen generation in groundwater OI-330.5, along with AHRL-69.4-O10 (*Planctomycetota*) and AHRL-69.4-O13 (VGIX01), contributing 2.7% and 1.7 to groundwater AHRL-69.4, respectively. Cryptic cycling of hydrogen in the AHRL-69.4 groundwater may have been partially mediated by AHRL-69.4-F6 (*Chloroflexota*) contributing 1.7% of the AHRL-69.4 hydrogen oxidation capacity. Finally, *Desulfobacterota* (AHRL-415.2-F1) was also suggested to play a key role in various metabolic processes in groundwater AHRL-200.6, including fermentation (0.8%), five classes of organic carbon oxidation (totaling 5.0%), acetate oxidation (1.8%), sulfur and sulfite oxidation (1.4% and 2.6%), and sulfate reduction (2.6%).

## Conclusions

The high number of novel FSG taxa corroborates the deep terrestrial biosphere as a reservoir of undiscovered microbial diversity that encodes a wide range of metabolic strategies to survive and significantly contribute to different processes in the ecosystem. These active novel taxa contributed to the metabolic potential for fermentation, amino acid utilization, complex carbon, aromatics, and fatty acid degradation, while expanding the metabolic capabilities of representatives in several phyla. The prevalence of the RNF complex in many novel taxa suggested that this may be an adaptation for energy conservation in low carbon and energy conditions. Community-level analysis revealed key contributions, particularly from representatives of the UBA9089 phylum, in biogeochemical processes. This exploration unveiled novel taxa with intriguing metabolic capabilities, offering valuable insights into deep biosphere microbial diversity and functional potential in emphasizing their significance in Fennoscandian Shield groundwater nutrient and energy cycling.

## Supplementary Material

Supplementary_files_ycae113

Supplementary_Table_S1_ycae113

Supplementary_Table_S2_ycae113

Supplementary_Table_S3_ycae113

Supplementary_Table_S4_ycae113

Supplementary_File_S2_ycae113

## Data Availability

Previously released FSG MAGs can be accessed through the NCBI BioProject under the accession number PRJNA627556. SAGs are publicly available in figshare with the identifier https://doi.org/10.6084/m9.figshare.12170313 under the project name “Fennoscandian Shield genomic database (FSGD).” The additional MAGs generated for this study are deposited under the BioProject accession number PRJNA1023754 and their accession numbers are given in Table 1 and Supplementary Table S1. A R Markdown file is available in Supplementary File S2.
