# Supplementary material for: Novel candidate taxa contribute to key metabolic processes in Fennoscandian Shield deep groundwaters
Source: ISME Commun. 2024 Sep 23;4(1):ycae113. doi: 10.1093/ismeco/ycae113 (PMC11484514; doi:10.1093/ismeco/ycae113)

Novel candidate taxa contribute to key metabolic processes in Fennoscandian Shield deep groundwaters

Mark Dopson, Maryam Rezaei Somee, Carolina González-Rosales, Lauren M Lui, Stephanie Turner,

Moritz Buck, Emelie Nilsson, George Westmeijer, Kamal Ashoor, Torben N Nielsen,

Maliheh Mehrshad, Stefan Bertilsson

Supplementary Data

Supplementary File S1

Nanopore sequencing and assembly

Nanopore libraries were prepared using the SQK-LSK109 kit (Oxford Nanopore Technologies, UK) and sequenced on a MinION with an R9.4.1 flow cell. The long-read assembly was done using Flye [1]. Other than specifying metagenomic mode, all parameters were left at their default settings.

Bioinformatic analyses

The metagenomic sequences were quality-checked and trimmed using Trimmomatic (version 0.36) (https://doi.org/10.1093/bioinformatics/btu170), where the Illumina TruSeq adapter was trimmed based on specific parameters ('TruSeq3-PE-2.fa:2:30:15 LEADING:3 TRAILING:3 SLIDINGWINDOW:4:15 MINLEN:31'). Subsequently, each dataset underwent individual assembly using MEGAHIT (version 1.1) (https://doi.org/10.1093/bioinformatics/btv033) with customized settings (--k-min 21 -k-max 141 --k-step 12 --min-count 2). Following assembly, contigs with a length of at least 2kb were automatically grouped using MetaBat2 (https://doi.org/10.7717/peerj.7359) with its default settings.

Fennoscandian Shield Genome (FSG) MAGs/SAGs with completeness ≥ 50% and contamination of ≤ 5% (using CheckM (v.1.2.0) [2]) were retained and their taxonomy was assigned via GTDB-tk v2.1.0 (reference database R207 [3]). A 95% average nucleotide identity (ANI) threshold was applied to dereplicate FSG MAGs/SAGs into metagenomic operational taxonomic units (mOTUs) using mOTUlizer (v.0.3.2 [4]). The general overview of the taxonomic distribution as per results of GTDB-tk analysis of the FSG MAG/SAG representatives was represented via a Sankey diagram, plotted using the Pavian package in R. Representative MAGs/SAGs, which were scored as unclassified at the family level and above by GTDB-tk, were selected for further analyses of novel taxa. This resulted in a total of 112 representative MAGs/SAGs that were assigned unique codes based on their origin with corresponding accession numbers given in Supplementary Table S2. Representative MAGs/SAGs that were denoted

as unclassified by GTDB-tk at family level and above (#112), were selected for further analysis. Each of these 112 representative MAGs/SAGs were assigned a unique code based upon the site where they were sampled with the corresponding accession numbers given in Supplementary Table S2.

The abundance of the 112 novel representative MAGs/SAGs was calculated using CoverM (v.0.6.1; https://github.com/wwood/CoverM) and by applying transcripts per million (TPM) as the normalization method. Moreover, abundance analyses were applied to FSG MAGs/SAGs, publicly available and published metagenomes, published MAGs, as well as SAGs originating from global deep oligotrophic terrestrial subsurface samples (≥70 m below the surface). METABOLIC-C v4.0 [5] in default settings was used to investigate the functional potential of the novel MAGs/SAGs and their contribution to metabolic processes in each borehole. From each borehole, a representative trimmed raw read sample was chosen for running METABOLIC in "community" mode. The MW-score (metabolic weight score) generated by the METABOLIC tool for each function in every borehole was plotted using the ggpubr package in R. The contribution of the candidate novel MAGs/SAGs at genome level were shown for the functions with MW-score ≥3 and plotted with pheatmap and complexheatmap packages in Rstudio. In addition, the novel MAGs/SAGs were initially annotated using PROKKA (v.1.12 [6]) and then functions were assigned using eggNOG-mapper (v.2.0.15 [7]). The resultant KEGG Orthologous information was used to extract *rnfABCDEG* genes presence/absence in the MAGs/SAGs.

RNA transcript-based activities of the representative MAGs/SAGs were analyzed in 9 metatranscriptomes (Table 1 & Supplementary Table S1) from Äspö HRL (KR0015, SA1420A, and SA2600A) boreholes. The transcripts were initially trimmed by bbduk.sh from BBMap toolkit (v.38.99-0 [8]). The abundance of 112 candidate MAGs/SAGs in each trimmed metatranscriptome sample was calculated by CoverM (v.0.6.1) using TPM as the normalization method (https://github.com/wwood/CoverM). The average TPM of nonzero samples was calculated per borehole and the abundance of 112 candidate MAGs/SAGs in metagenomes and metatranscriptome samples per borehole were plotted with pheatmap and complexheatmap packages in Rstudio.

References

1. Kolmogorov, M, DM Bickhart, B Behsaz, A Gurevich, M Rayko, SB Shin, et al. metaFlye: scalable long-read metagenome assembly using repeat graphs. Nat Meth. 2020;17:1103-1110.
2. Parks, DH, M Imelfort, CT Skennerton, P Hugenholtz, GW Tyson. CheckM: assessing the quality of microbial genomes recovered from isolates, single cells, and metagenomes. Genome Res. 2015;25:1043-1055.
3. Chaumeil, P-A, AJ Mussig, P Hugenholtz, DH Parks. GTDB-Tk v2: memory friendly classification with the genome taxonomy database. Bioinformatics. 2022;38:5315-5316.
4. Buck, M, M Mehrshad, S Bertilsson. mOTUpan: a robust Bayesian approach to leverage metagenome-assembled genomes for core-genome estimation. NAR Genomics and Bioinformatics. 2022;4.
5. Zhou, Z, PQ Tran, AM Breister, Y Liu, K Kieft, ES Cowley, et al. METABOLIC: high-throughput profiling of microbial genomes for functional traits, metabolism, biogeochemistry, and community-scale functional networks. Microbiome. 2022;10:33.
6. Seemann, T. Prokka: rapid prokaryotic genome annotation. Bioinformatics. 2014;30:2068-2069.
7. Cantalapiedra, CP, A Hernández-Plaza, I Letunic, P Bork, J Huerta-Cepas. eggNOG-mapper v2: Functional annotation, orthology assignments, and domain prediction at the metagenomic scale. Mol Biol Evol. 2021;38:5825-5829.
8. Bushnell, B, *BBMap: A fast, accurate, splice-aware aligner*. 2014: Lawrence Berkeley National Laboratory. p. https://escholarship.org/uc/item/1h3515gn.

Supplementary File S2. A R file with the code for data analysis has been uploaded.

Supplementary Table S1. Details of sampled groundwaters, and accession numbers for (meta)genomes and metatranscriptomes not previously published.

A separate Excel file has been uploaded.

Supplementary Table S2. Details of MAGs and SAGs used in the present study along with the genomes sorted for the novel Fennoscandian Shield Genome taxa.

A separate Excel file has been uploaded.

Supplementary Table S3. Coverage of all MAGs in the community metagenome and metatranscriptome along with the contribution of the 112 novel taxa in each of the groundwaters.

A separate Excel file has been uploaded.

Supplementary Table S4. Contribution of the 112 novel taxa in each METABOLIC function expressed as a percentage of the total community.

A separate Excel file has been uploaded.

Supplementary Figure S1. Presence of metabolic traits in the novel class, order, and family Fennoscandian Shield Genomes.

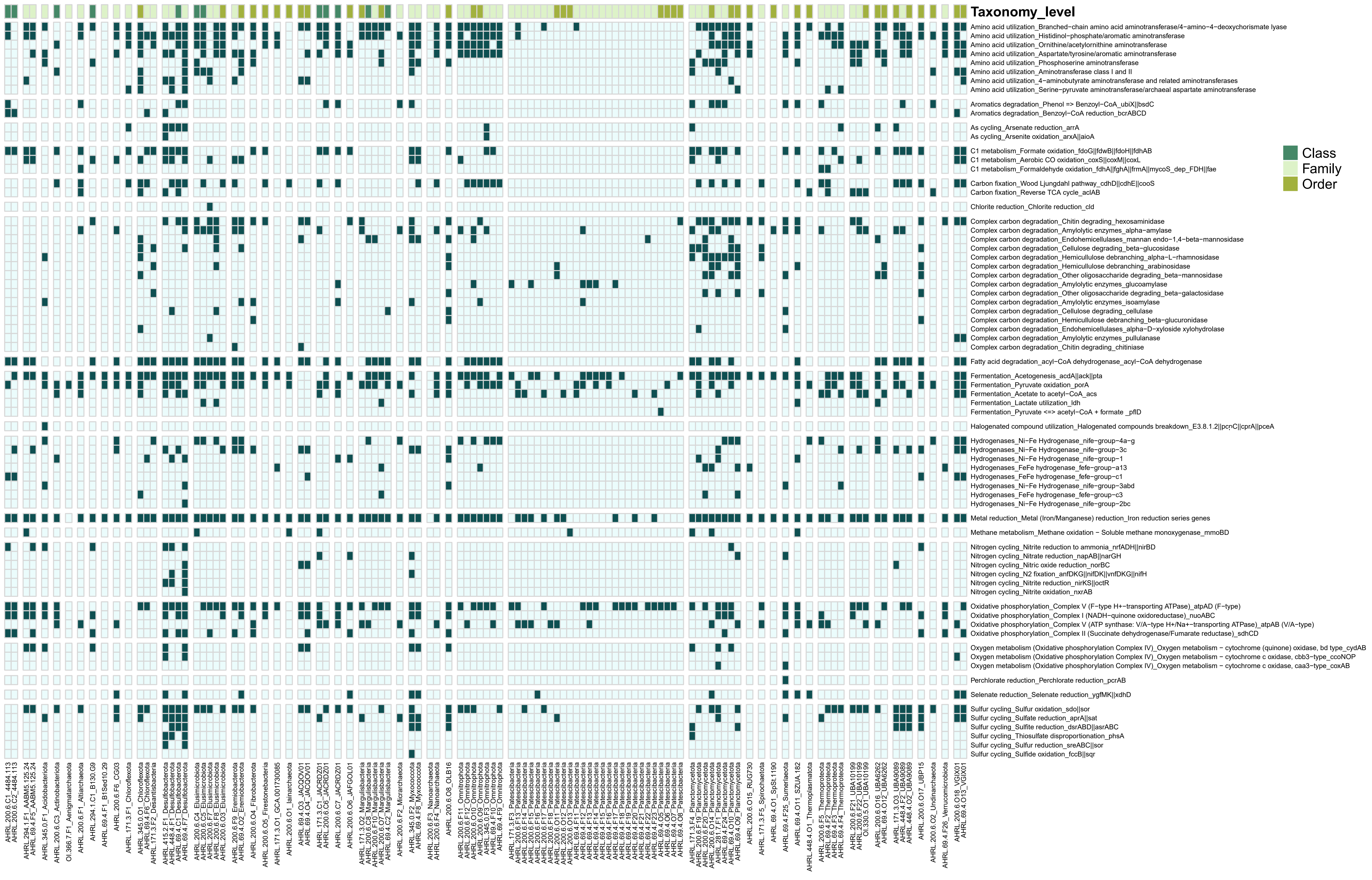

Taxonomy_level
Class
Family
Order
Amino acid utilization_Branched−chain amino acid aminotransferase/4−amino−4−deoxychorismate lyase
Amino acid utilization_Histidinol−phosphate/aromatic aminotransferase
Amino acid utilization_Ornithine/acetylornithine aminotransferase
Amino acid utilization_Aspartate/tyrosine/aromatic aminotransferase
Amino acid utilization_Phosphoserine aminotransferase
Amino acid utilization_Aminotransferase class I and II
Amino acid utilization_4−aminobutyrate aminotransferase and related aminotransferases
Amino acid utilization_Serine−pyruvate aminotransferase/archaeal aspartate aminotransferase
Aromatics degradation_Phenol => Benzoyl−CoA_ubiX||bsdC
Aromatics degradation_Benzoyl−CoA reduction_bcrABCD
As cycling_Arsenate reduction_arrA
As cycling_Arsenite oxidation_arxA||aioA
C1 metabolism_Formate oxidation_fdoG||fdwB||fdoH||fdhAB
C1 metabolism_Aerobic CO oxidation_coxS||coxM||coxL
C1 metabolism_Formaldehyde oxidation_fdhA||fghA||frmA||mycoS_dep_FDH||fae
Carbon fixation_Wood Ljungdahl pathway_cdhD||cdhE||cooS
Carbon fixation_Reverse TCA cycle_aclAB
Chlorite reduction_Chlorite reduction_cld
Complex carbon degradation_Chitin degrading_hexosaminidase
Complex carbon degradation_Amylolytic enzymes_alpha−amylase
Complex carbon degradation_Endohemicellulases_mannan endo−1,4−beta−mannosidase
Complex carbon degradation_Cellulose degrading_beta−glucosidase
Complex carbon degradation_Hemicellulose debranching_alpha−L−rhamnosidase
Complex carbon degradation_Hemicellulose debranching_arabinosidase
Complex carbon degradation_Other oligosaccharide degrading_beta−mannosidase
Complex carbon degradation_Amylolytic enzymes_glucoamylase
Complex carbon degradation_Other oligosaccharide degrading_beta−galactosidase
Complex carbon degradation_Amylolytic enzymes_isoamylase
Complex carbon degradation_Cellulose degrading_cellulase
Complex carbon degradation_Hemicellulose debranching_beta−glucuronidase
Complex carbon degradation_Endohemicellulases_alpha−D−xyloside xylohydrolase
Complex carbon degradation_Amylolytic enzymes_pullulanase
Complex carbon degradation_Chitin degrading_chitinase
Fatty acid degradation_acyl−CoA dehydrogenase_acyl−CoA dehydrogenase
Fermentation_Acetogenesis_acdA||ack||pta
Fermentation_Pyruvate oxidation_porA
Fermentation_Acetate to acetyl−CoA_acs
Fermentation_Lactate utilization_ldh
Fermentation_Pyruvate <=> acetyl−CoA + formate _pflD
Halogenated compound utilization_Halogenated compounds breakdown_E3.8.1.2||pcpC||cprA||pceA
Hydrogenases_Ni−Fe Hydrogenase_nife−group−4a−g
Hydrogenases_Ni−Fe Hydrogenase_nife−group−3c
Hydrogenases_Ni−Fe Hydrogenase_nife−group−1
Hydrogenases_FeFe hydrogenase_fefe−group−a13
Hydrogenases_FeFe hydrogenase_fefe−group−c1
Hydrogenases_Ni−Fe Hydrogenase_nife−group−3abd
Hydrogenases_FeFe hydrogenase_fefe−group−c3
Hydrogenases_Ni−Fe Hydrogenase_nife−group−2bc
Metal reduction_Metal (Iron/Manganese) reduction_Iron reduction series genes
Methane metabolism_Methane oxidation − Soluble methane monoxygenase_mmoBD
Nitrogen cycling_Nitrite reduction to ammonia_nrfADH||nirBD
Nitrogen cycling_Nitrate reduction_napAB||narGH
Nitrogen cycling_Nitric oxide reduction_norBC
Nitrogen cycling_N2 fixation_anfDKG||nifDK||vnfDKG||nifH
Nitrogen cycling_Nitrite reduction_nirKS||octR
Nitrogen cycling_Nitrite oxidation_nxrAB
Oxidative phosphorylation_Complex V (F−type H+−transporting ATPase)_atpAD (F−type)
Oxidative phosphorylation_Complex I (NADH−quinone oxidoreductase)_nuoABC
Oxidative phosphorylation_Complex V (ATP synthase: V/A−type H+/Na+−transporting ATPase)_atpAB (V/A−type)
Oxidative phosphorylation_Complex II (Succinate dehydrogenase/Fumarate reductase)_sdhCD
Oxygen metabolism (Oxidative phosphorylation Complex IV)_Oxygen metabolism − cytochrome (quinone) oxidase, bd type_cydAB
Oxygen metabolism (Oxidative phosphorylation Complex IV)_Oxygen metabolism − cytochrome c oxidase, cbb3−type_ccoNOP
Oxygen metabolism (Oxidative phosphorylation Complex IV)_Oxygen metabolism − cytochrome c oxidase, caa3−type_coxAB
Perchlorate reduction_Perchlorate reduction_pcrAB
Selenate reduction_Selenate reduction_ygfMK||xdhD
Sulfur cycling_Sulfur oxidation_sdo||sor
Sulfur cycling_Sulfate reduction_aprA||sat
Sulfur cycling_Sulfite reduction_dsrABD||asrABC
Sulfur cycling_Thiosulfate disproportionation_phsA
Sulfur cycling_Sulfur reduction_sreABC||sor
Sulfur cycling_Sulfide oxidation_fccB||sqr
AHRL.200.6.C1_4484.113
AHRL.200.6.C2_4484.113
AHRL.294.1.F1_AABM5.125.24
AHRL.69.4.F5_AABM5.125.24
AHRL.345.0.F1_Acidobacteriota
AHRL.200.6.C3_Actinobacteriota
OI.366.7.F1_Aenigmatarchaeota
AHRL.200.6.F1_Altiarchaeota
AHRL.294.1.C1_B130.G9
AHRL.69.4.F1_B1Sed10.29
AHRL.200.6.F6_CG03
AHRL.171.3.F1_Chloroflexota
AHRL.345.0.O1_Chloroflexota
AHRL.69.4.F6_Chloroflexota
AHRL.171.3.F2_Desantisbacteria
AHRL.415.2.F1_Desulfobacterota
AHRL.448.4.F1_Desulfobacterota
AHRL.69.4.C1_Desulfobacterota
AHRL.69.4.F7_Desulfobacterota
AHRL.200.6.C4_Elusimicrobiota
AHRL.200.6.C5_Elusimicrobiota
AHRL.200.6.F7_Elusimicrobiota
AHRL.200.6.F8_Elusimicrobiota
AHRL.200.6.O3_Elusimicrobiota
AHRL.200.6.F9_Eremiobacterota
AHRL.69.4.O2_Eremiobacterota
AHRL.200.6.O4_Fibrobacterota
AHRL.200.6.O5_Firestonebacteria
AHRL.171.3.O1_GCA.001730085
AHRL.200.6.O1_Iainarchaeota
AHRL.69.4.O3_JACQOV01
AHRL.69.4.O4_JACQOV01
AHRL.171.3.C1_JACRDZ01
AHRL.200.6.C6_JACRDZ01
AHRL.200.6.C7_JACRDZ01
AHRL.200.6.O6_JAFGOL01
AHRL.171.3.O2_Margulisbacteria
AHRL.200.6.C8_Margulisbacteria
AHRL.200.6.F10_Margulisbacteria
AHRL.200.6.O7_Margulisbacteria
AHRL.69.4.C2_Margulisbacteria
AHRL.200.6.F2_Micrarchaeota
AHRL.345.0.F2_Myxococcota
AHRL.69.4.F8_Myxococcota
AHRL.200.6.F3_Nanoarchaeota
AHRL.200.6.F4_Nanoarchaeota
AHRL.200.6.O8_OLB16
AHRL.200.6.F11_Omnitrophota
AHRL.200.6.F12_Omnitrophota
AHRL.200.6.O10_Omnitrophota
AHRL.200.6.O9_Omnitrophota
AHRL.345.0.F3_Omnitrophota
AHRL.69.4.F10_Omnitrophota
AHRL.69.4.F9_Omnitrophota
AHRL.171.3.F3_Patescibacteria
AHRL.200.6.F13_Patescibacteria
AHRL.200.6.F14_Patescibacteria
AHRL.200.6.F15_Patescibacteria
AHRL.200.6.F16_Patescibacteria
AHRL.200.6.F17_Patescibacteria
AHRL.200.6.F18_Patescibacteria
AHRL.200.6.O11_Patescibacteria
AHRL.200.6.O12_Patescibacteria
AHRL.200.6.O13_Patescibacteria
AHRL.69.4.F11_Patescibacteria
AHRL.69.4.F12_Patescibacteria
AHRL.69.4.F13_Patescibacteria
AHRL.69.4.F14_Patescibacteria
AHRL.69.4.F15_Patescibacteria
AHRL.69.4.F16_Patescibacteria
AHRL.69.4.F17_Patescibacteria
AHRL.69.4.F18_Patescibacteria
AHRL.69.4.F19_Patescibacteria
AHRL.69.4.F20_Patescibacteria
AHRL.69.4.F21_Patescibacteria
AHRL.69.4.F22_Patescibacteria
AHRL.69.4.F23_Patescibacteria
AHRL.69.4.O5_Patescibacteria
AHRL.69.4.O6_Patescibacteria
AHRL.69.4.O7_Patescibacteria
AHRL.69.4.O8_Patescibacteria
AHRL.171.3.F4_Planctomycetota
AHRL.200.6.F19_Planctomycetota
AHRL.200.6.F20_Planctomycetota
AHRL.200.6.O14_Planctomycetota
AHRL.281.7.F1_Planctomycetota
AHRL.69.4.F24_Planctomycetota
AHRL.69.4.O10_Planctomycetota
AHRL.69.4.O9_Planctomycetota
AHRL.200.6.O15_RUG730
AHRL.171.3.F5_Spirochaetota
AHRL.69.4.O1_SpSt.1190
AHRL.69.4.F25_Sumerlaeota
AHRL.69.4.O11_SZUA.182
AHRL.448.4.O1_Thermoplasmatota
AHRL.200.6.F5_Thermoproteota
AHRL.69.4.F2_Thermoproteota
AHRL.69.4.F3_Thermoproteota
AHRL.69.4.F4_Thermoproteota
AHRL.200.6.F21_UBA10199
AHRL.200.6.F22_UBA10199
OI.330.5.O1_UBA10199
AHRL.200.6.O16_UBA6262
AHRL.69.4.O12_UBA6262
AHRL.171.3.O3_UBA9089
AHRL.448.4.F2_UBA9089
AHRL.448.4.O2_UBA9089
AHRL.200.6.O17_UBP15
AHRL.200.6.O2_Undinarchaeota
AHRL.69.4.F26_Verrucomicrobiota
AHRL.200.6.O18_VGIX01
AHRL.69.4.O13_VGIX01

Supplementary Figure S2. Presence of *rnf* complex genes in the novel class, order, and family Fennoscandian Shield Genomes.

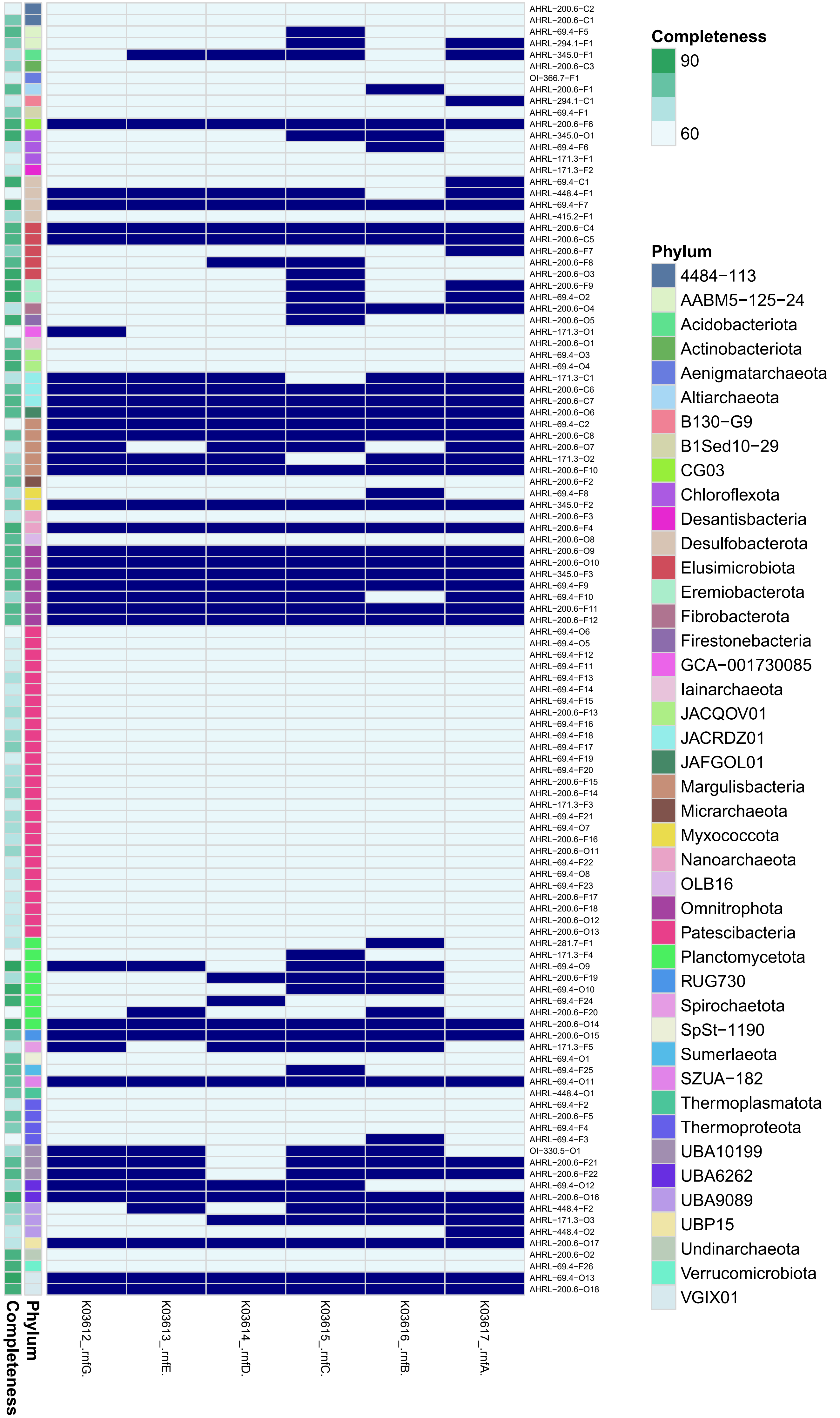

Completeness
90
60
Phylum
4484−113
AABM5−125−24
Acidobacteriota
Actinobacteriota
Aenigmatarchaeota
Altiarchaeota
B130−G9
B1Sed10−29
CG03
Chloroflexota
Desantisbacteria
Desulfobacterota
Elusimicrobiota
Eremiobacterota
Fibrobacterota
Firestonebacteria
GCA−001730085
Iainarchaeota
JACQOV01
JACRDZ01
JAFGOL01
Margulisbacteria
Micrarchaeota
Myxococcota
Nanoarchaeota
OLB16
Omnitrophota
Patescibacteria
Planctomycetota
RUG730
Spirochaetota
SpSt−1190
Sumerlaeota
SZUA−182
Thermoplasmatota
Thermoproteota
UBA10199
UBA6262
UBA9089
UBP15
Undinarchaeota
Verrucomicrobiota
VGIX01
K03612_rnfG.
K03613_rnfE.
K03614_rnfD.
K03615_rnfC.
K03616_rnfB.
K03617_rnfA.

Supplementary Figure S3. Metabolic weight score of biochemical functions comes from METABOLIC-C analysis calculated based on all de-replicated MAGs from the Fennoscandian Shield Genomes metabolic potential for each borehole. Circle size and color scale both represent the percentage contribution of each MAG in the respective metabolic modules.

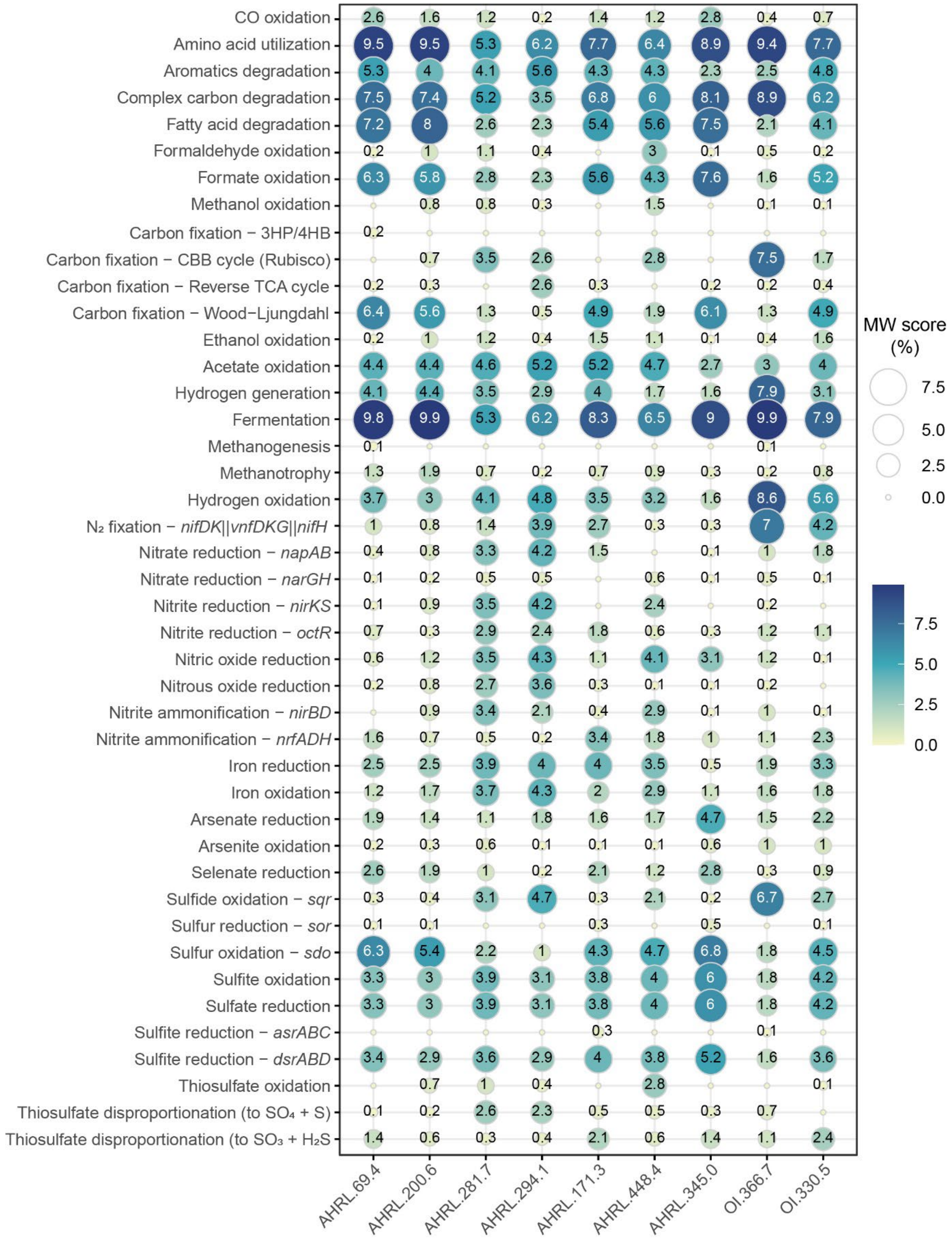

Supplement: Supplementary_files_ycae113 [file supplementary_files_ycae113.pdf]
